# The Factorial Structure of the Amsterdam Preoperative Anxiety and Information Scale in Parents of Pediatric Surgical Patients: An Item Response Theory Analysis

**DOI:** 10.1002/brb3.71760

**Published:** 2026-09-14

**Authors:** Brigitte Messerer, Carmen Burger‐Ringer, Marko Stijic, Alexander Avian

**Affiliations:** ^1^ Department of Anaesthesiology and Intensive Care Medicine Medical University of Graz Graz Austria; ^2^ Institute for Medical Informatics, Statistics and Documentation Medical University of Graz Graz Austria

**Keywords:** factorial structure, preoperative anxiety, preoperative need for information, reliability

## Abstract

**Background:**

Preoperative anxiety is a common experience among patients undergoing surgery. The Amsterdam Preoperative Anxiety and Information Scale (APAIS) is a short questionnaire designed to assess anxiety related to anesthesia and surgery, as well as patient's need for preoperative information (anesthesia and surgery). Despite its widespread use, comprehensive psychometric analyses using modern latent variable models remain limited.

**Methods:**

In this cross‐sectional psychometric analysis nested within a larger observational study, parents of children undergoing surgery answered the APAIS before their children's surgery. Responses were analyzed using item response theory (IRT) methods.

**Results:**

Of the 477 parents who participated in the survey, 476 completed the APAIS. Participant's age ranged from 24 to 65 years (female: 76.1%). None of the tested models (one‐ to three‐factor structures using different IRT‐models) showed acceptable fit across all indices (root mean square error of approximation [RMSEA] < 0.08, Tucker–Lewis index [TLI] ≥ 0.90, comparative fit index [CFI] ≥ 0.90). For the originally proposed factorial structure using a graded response model (RMSEA: 0.26, TLI: 0.82, CFI: 0.91), reliability was high (*r* > 0.8) for the middle area of −1.4 to +2.7 logits for anxiety and −1.4 to +1.8 logits for need for information. The magnitude of all statistically significant differential item functioning (DIF) effects was below the predefined threshold for clinical relevance.

**Conclusions:**

None of the previously proposed factorial structures met the criteria for good model fit. The best‐fitting model was the original two‐factor structure, which showed poor model fit regarding RMSEA and acceptable model fit regarding CFI for all test models. TLI was acceptable for the partial credit model only. Further studies with larger sample sizes and the application of modern psychometric methods, such as IRT, are needed to clarify the factorial structure of the APAIS.

## Graphical abstract

In the analysis of the Amsterdam Operative Anxiety and Information Scale (APAIS) using Item Response Theory, no previously published structure showed a good model fit. The original two‐factor structure showed the best fit. In this solution, the factors also demonstrated high reliability across a wide range of the measured latent variables.

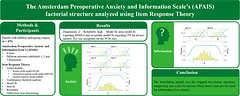

**Abbreviations**
AIC: Akaike information criterion
APAIS: Amsterdam Preoperative Anxiety and Information Scale
BIC: Bayesian information criterion
CFA: confirmatory factor analysis
CFI: comparative fit index
df: degrees of freedom
DIF: differential item functioning
GPCM: generalized partial credit model
GRM: graded response model
IQR: interquartile range
IRT: item response theory
logLik: log‐likelihood
PCM: partial credit model
RMSEA: root mean square error of approximation
SABIC: sample‐size adjusted BIC
STADI: State–Trait–Anxiety–Depression Inventory
TLI: Tucker–Lewis index

## 1 Introduction

Preoperative anxiety is common among surgical patients. A meta‐analysis of 28 studies including 14,652 patients reported a pooled prevalence of 48%, with estimates ranging from 17% to 88% across individual studies (Abate et al. 2020). Preoperative anxiety impacts anesthetic and analgesic requirements, risk for delirium, and sleep quality, and is therefore an important factor in postoperative recovery (Shebl et al. 2025; Gu et al. 2023). Therefore, reliable measurement of preoperative anxiety is essential for both clinical practice and research. A frequently used tool for this purpose is the Amsterdam Preoperative Anxiety and Information Scale (APAIS) (Moerman et al. 1996). This tool has gained broad acceptance due to very reliable measurements with just a few items. In both routine clinical practice and clinical trials, the use of concise instruments is particularly important to minimize the burden on patients and avoid disrupting the clinical workflow.

The APAIS consist of six items: four assessing anxiety related to anesthesia and surgery, and two assessing the need for information regarding these domains. The original publication by Moerman et al. (1996) suggested a two‐factor structure (anxiety and need for information), which was also supported by other studies (Wu et al. 2020). However, subsequent research has reported alternative structures, including a single‐factor model (Vergara‐Romero et al. 2017; Farooqui et al. 2023), a two‐factor model distinguishing surgery and anesthesia (*F*1: surgery, *F*2: anesthesia) (Jovanovic et al. 2022), and a three‐factor model (*F*1: surgery anxiety, *F*2: anesthesia anxiety, *F*3: need for information) (Maurice‐Szamburski et al. 2013; Maurício et al. 2021; Srinivasaiah et al. 2024). Regardless of the factor structure identified, all models demonstrated acceptable to very good internal consistency, as measured by Cronbach's *α*.

Although these results appear promising, they do not take into account the fact that the accuracy of measurements depends on the level of the latent variable being measured (Embretson and Reise 2000). Therefore, reliability is highest at moderate levels (e.g., moderate anxiety) and lower at the extremes (no anxiety or high anxiety). When reliability is summarized using a single coefficient (e.g., Cronbach's *α*), this variation is not captured. Another aspect that was investigated in only one study is measurement invariance (differential item functioning [DIF]). Measurement invariance describes an item's ability to possess the same properties across different groups. Maurice‐Szamburski et al. (2013) reported only a small amount of DIF regarding age, gender, type of anesthesia or surgery, premedication, ASA (American Society of Anesthesiologists) physical status, and ambulatory course). Both reliability as a function of the level of the measured latent variable and measurement invariance can be analyzed using item response theory (IRT) methods. However, IRT methods were only used once to analyze the APAIS (Maurice‐Szamburski et al. 2013). Another frequently overlooked aspect is the extent to which the proposed models (e.g., factorial structures) adequately fit the underlying data. Only studies reporting confirmatory factor analysis (CFA) (Wu et al. 2020; Vergara‐Romero et al. 2017; Jovanovic et al. 2022; Maurice‐Szamburski et al. 2013; Maurício et al. 2021; Srinivasaiah et al. 2024) reported model fit indices, whereas the majority of published studies relied on exploratory factor analysis (Farooqui et al. 2023; ALMesned et al. 2022; Berth et al. 2007; Buonanno et al. 2017; Çetinkaya et al. 2019; Nishimori et al. 2002; Miraglia Raineri et al. 2019; Mohd Fahmi et al. 2015; Zeleníková et al. 2017) without reporting model fit.

Despite the widespread use of the APAIS, comprehensive psychometric analyses using modern latent variable models are lacking. In particular, systematic investigations of measurement invariance and trait‐dependent reliability of the scale are lacking. Therefore, the aim of this secondary analysis is to evaluate APAIS responses in a setting that has not been studied before. Parents of children undergoing surgeries were included in a larger observational study in which parental preoperative anxiety regarding the child's surgery was assessed. Using IRT methods including the assessment of DIF and trait‐dependent reliability, evaluation of the factor structure of the APAIS and the overall model fit parent's responses are evaluated.

## 2 Methods

This cross‐sectional psychometric analysis nested within a larger observational study was conducted in accordance with the Declaration of Helsinki (World Medical Association). The study protocol was approved by the local ethics committee, Medical University of Graz, Austria (Ethic Committee number: 32–157 ex 19/20). The present study was part of a larger research project investigating various aspects of children's hospital stay, including side effects (e.g., pain, postoperative nausea, and vomiting), sleep, and the impact of parental characteristics. In the current analysis, parents completed the APAIS to investigate whether parental anxiety influences the child's hospital experience. Parents were approached in the waiting room by a trained psychologist or a medical student, and after providing informed consent, they completed the questionnaires. This study was conducted from October 7, 2022 to January 31, 2025 at the Department of Pediatric and Adolescent Surgery at the University Hospital of Graz, Austria. Inclusion criteria were (1) ability to speak German, (2) child undergoing a surgical procedure, (3) absence of cognitive impairment in the child, (4) child aged between 4 and 18 years, and (5) provision of written informed consent. Parents/children who have previously participated in this study were not included.

One parent of each child completed an online LimeSurvey questionnaire providing information on their demographic characteristics (sex, age, educational level, relationship status, number of children), as well as psychological variables including state and trait anxiety, depressive symptoms, prior experience as a patient, coping styles, and their child's previous surgical experiences.

### 2.1 Materials

The APAIS consist of six items originally grouped into two scales (anxiety: four items; need for information: two items). Items are answered using a 5‐point Likert‐type response scale from 1 “not at all” to 5 “extremely.” Using traditional classical test theory scales, scale scores are calculated by summing up the individual responses. These results are in a range of 4–20 for the anxiety scale and 2–10 for the need for information scale. Wording of these scales were not changed for this study.

Parents’ state and trait anxiety as well as their depressive symptoms were assessed using the 20‐item State–Trait–Anxiety–Depression Inventory (STADI) which contains four subscales (anxiety—emotional, anxiety—worry, depression—anhedonia, depression—dysthymia) (Laux et al. 2013). Responses are recorded on Likert‐type scales ranging from 1 (almost never) to 4 (almost always). The global trait scale shows good test–retest reliability for 1 week (*r* = 0.85), 4 weeks (*r* = 0.87), and 14 months (*r* = 0.85). As expected, the state scale shows lower test–retest reliability over 1 week (*r* = 0.35), 4 weeks (*r* = 0.56), and 14 months (*r* = 0.20). Internal consistency was high for the global trait scale (Cronbach's *α*: 0.93), trait subscales (Cronbach's *α*: 0.81–0.89), global state scale (Cronbach's *α*: 0.92), and state subscales (Cronbach's *α*: 0.83–0.90).

Coping styles were assessed using the German adaptation of the Coping Inventory for Stressful Situations (CISS) (Kälin and Semmer 2020), a shortened 24‐item version of the original 48‐item instrument. The questionnaire comprises three main subscales: task‐oriented coping, emotion‐oriented coping, and avoidance‐oriented coping. The latter includes two sub‐subscales: social diversion and distraction. Internal consistency coefficients for the subscales ranged from Cronbach's *α* = 0.63 (distraction) to *α* = 0.83 (social diversion). Test–retest reliability, assessed in a sample of young working Swiss adults (*N* = 359) over a 1‐year interval, ranged from *r* = 0.47 (distraction‐oriented coping) to *r* = 0.65 (emotion‐oriented coping).

Parental experience as a patient was assessed using the 15‐item Picker Patient Experience Questionnaire (PPE‐15) (Jenkinson et al. 2002). The PPE‐15 was developed to collect patient feedback, identify areas of care in need of improvement, and monitor the performance of the health care system. It comprises 15 items covering seven aspects of care: respect, coordination, information/communication/education, physical comfort, emotional support, involvement of relatives, and transitions and continuity. The items have two (“yes” or “no”) to four response options (“yes,” “no,” “I did not need to,” or “yes, to some extent”). Internal consistency coefficient for the total scale in German sample was *r* = 0.85 over a 1‐month interval.

### 2.2 Data Analysis

Categorical data are presented as absolute and relative frequencies, and continuous data as means and standard deviations or medians with interquartile ranges (IQR), as appropriate. In the first step, category response frequencies were analyzed. In the second step, IRT‐based analysis were performed. Three models suitable for polytomous responses—the partial credit model (PCM), the generalized PCM (GPCM), and the graded response model (GRM)—were compared using likelihood ratio test for nested models (PCM vs. GPCM) and Voung test for comparison of non‐nested models (PCM vs. GRM, GPCM vs. GRM) to find the model best fitting to the data. These three models differ in whether a discrimination parameter is included and whether the response categories must be ordered. To identify the optimal factor structure within the selected IRT model, previously published factorial structures were compared using likelihood ratio tests. Therefore, unidimensional models (Model 1), two‐dimensional models from the original publication (Model 2a: anxiety about anesthesia and surgery/need for information), an alternative two‐dimensional model (Model 2b: anxiety and need for information about anesthesia/anxiety and need for information about surgery), and a three‐dimensional model (Model 3: anxiety about anesthesia/anxiety about surgery/need for information) were compared using likelihood ratio tests. To assess the model fit for all models, comparative fit index (CFI ≥ 0.90), Tucker–Lewis index (TLI ≥ 0.90), and root mean square error of approximation (RMSEA < 0.08) were calculated. To make use of all available information for the calculation of fit indices, missing responses were imputed based on the estimated latent trait. Imputation was done 1.000 times and mean CFI, TLI, and RMSEA were calculated. Estimated item parameters (difficulty and discrimination) of the final model were reported, and a Wright map was created to compare item difficulty and participant ability. To examine floor and ceiling effects the proportion of participants below the lowest threshold and above the highest threshold was calculated for each item.

Furthermore, item characteristic curves are given. Reliability was calculated and depending on the ability level graphically shown. Reliability > 0.8 was considered as sufficient. For analysis of measurement invariance, DIF analysis for sex (male vs. female), age (≤ 40 years, > 40 years), educational level (high school degree: yes/no), relationship status (married: yes/no), number of children (1 vs. ≥ 2), and children's previous surgical experiences (surgery before: yes/no) were performed. To determine whether measurement invariance was present, a significant result was required, and the magnitude of the DIF had to be Δ*R* ^2^ ≥ 0.13 or Δ*β* ≥ 0.1. Since it was observed, that only relying on *p*‐value for DIF detection also very small, DIF could be flagged; also the criteria proposed by Zumbo (1999) for Δ*R* ^2^ (negligible: < 0.13; moderate: between 0:13 and 0:26; large: > 0.26) and Crane et al. (2004) for Δ*β* was used.

To get some insight in validity, associations with other questionnaires were analyzed. For scales of the final model including anxiety items, positive correlations are expected with STADI scales, with higher correlation coefficients for state compared to trait scales. No meaningful correlations are expected with coping styles or parental experience as a patient. If the final model results in a factor only including information items, no meaningful correlation with any of the questionnaires used is to be expected. To decide whether a correlation is meaningful the thresholds of Cohen are used (small: *r* = 0.20, medium: *r* = 0.50, large: *r* = 0.80) (Cohen 1988). Therefore at least medium correlations (*r* > 0.50) are considered meaningful. Statistical analysis was performed using R studio version 4.5.0 using the packages mirt (Chalmers 2012) and lordif (Choi et al. 2011).

## 3 Result

### 3.1 Study Participants

Of the 477 parents who completed the survey, 476 responded to the APAIS. Parents' ages ranged from 24 to 65 (median: 41; IQR: 36–46; missing values: *n* = 31). The majority were female (76.1%; missing values: *n* = 11), did not have a high school degree (54.2%), were married (73.1%), and had at least two children (80%) (Table 1).

**TABLE 1:** Demographic parameters of the participants.

Characteristic		*n* (%)
Sex	Male	103 (21.6)
	Female	362 (76.1)
	Diverse	0 (—)
	Missing	11 (2.3)
Age	≤ 40 years	217 (45.6)
	> 40 years	228 (47.9)
	Missing	31 (6.5)
Educational level (high school degree)	Yes	207 (43.5)
	No	258 (54.2)
	Missing	11 (2.3)
Relationship status (married)	Yes	348 (73.1)
	No	128 (26.9)
Number of children	1	84 (17.6)
	≥ 2	381 (80.0)
	Missing	11 (2.3)
Children's preexisting experiences with surgeries	Yes	247 (51.9)
	No	229 (48.1)
Children's surgery	Bones	136 (28.6)
	Urogenital tract	108 (22.7)
	Ear, nose, and throat	99 (20.8)
	Minor surgery	52 (10.9)
	Plastic surgery	28 (5.9)
	Inguinal surgery	17 (3.6)
	Investigations	11 (2.3)
	Knee, shoulder, hip, joints	7 (1.5)
	Teeth	7 (1.5)
	Abdominal procedures	5 (1.1)
	Missing	6 (1.3)
ASA status	1	371 (77.9)
	2	96 (20.2)
	3	3 (0.6)
	Missing	5 (1.1)
Anesthesia	General anesthesia	262 (55.0)
	General and regional anesthesia	201 (42.2)
	Analgesic sedation	5 (1.1)
	Missing	8 (1.7)

### 3.2 Response Patterns

**FIGURE 1:**
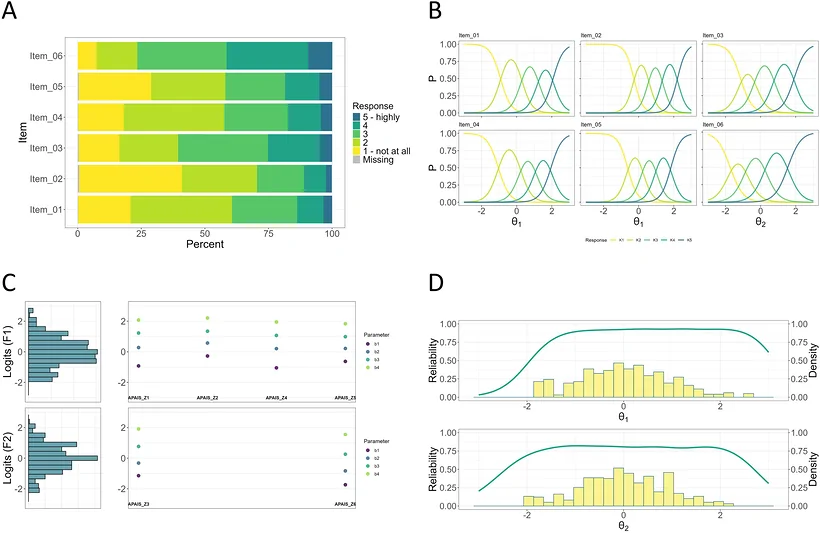
Results of the psychometric analysis. (A) Response pattern for the original six items. (B) Item characteristic curves for the six items: two‐dimensional 2PL model. (C) Wright map for both factors. (D) Two‐dimensional reliability surface. Threshold of reliability = 0.8 is given with a black line.

The items had a maximum of 0.6% missing values (“APAIS_02”). Most responses were at the lower end of the scale (“not at all”), chosen by 7.4%–40.3% of respondents. In contrast, the highest response category (“highly”) was selected by only 2.3%–9.5% of respondents (Figure 1A and Table 2).

**TABLE 2:** Response frequencies.

		1—Not at all	2	3	4	5—Highly	Missing
Item_01	I am worried about the anesthesia.	20.6	39.9	25.6	10.3	3.4	0.2
Item_02	The anesthetic is on my mind continually.	40.3	29.6	18.5	8.6	2.3	0.6
Item_03	I would like to know as much as possible about the anesthetic.	16.0	23.1	35.3	20.4	4.8	0.4
Item_04	I am worried about the procedure.	17.9	39.5	25.0	13.0	4.4	0.2
Item_05	The procedure is on my mind continually.	28.4	29.2	23.5	13.5	5.0	0.4
Item_06	I would like to know as much as possible about the procedure.	7.4	16.0	35.1	32.1	9.5	0.0

### 3.3 Psychometric Properties

None of the analyzed models showed acceptable fit indices for all reported fit indices (RMSEA < 0.08, TLI ≥ 0.9, CFI ≥ 0.9) (Table 3). The RMSEA was not acceptable for any model. Using the PCM showed acceptable CFI and TLI values for the original proposed structure. The GPCM and the GRM, showed acceptable CFI and TLI slightly below predefine threshold of 0.9 for both two‐dimensional models. Since the originally proposed structure achieved an acceptable model fit for at least one fit index in all analyzed IRT models, and the lowest relative fit indices (Akaike information criterion [AIC], Bayesian information criterion [BIC], and sample‐size adjusted BIC [SABIC]) were observed when using the GRM, the results for this model are presented in detail. This model showed acceptable fit according to TLI (0.82) but poor model fit according to CFI (0.91) and RMSEA (0.24, 90% CI: 0.21–0.26). Factor loadings of each item to the corresponding factor were high (> 0.845). Discrimination parameter (*a*_1_, *a*_2_) and threshold parameters (*b*_1_, *b*_2_, *b*_3_, *b*_4_) are reported in Table 4 and item characteristic curves are shown in Figure 1B. For both factors, threshold parameters covered a wide range of measured latent variable (*F*1: −0.92 to 2.21, *F*2: −1.74 to 1.95) corresponding well with the respondents’ latent trait distribution (Figure 1C). The median anxiety score was −0.03 (IQR: −0.67 to 0.67; range: −1.85 to 2.63). The median need for information was −0.03 (IQR: −0.60 to 0.60; range: −2.02 to 2.28). The two factors showed a moderate positive correlation of *r* = 0.583.

**TABLE 3:** Model fit for published factorial structures using PCM, GPCM, and GRM for modeling. Acceptable values for model fit are given in bold.

Model	logLik	df	AIC	BIC	SABIC	RMSEA (90% CI)	TLI	CFI
PCM—Dim 1	−3452.07	15,599	6954.15	7058.29	6978.94	0.30 (0.28–0.32)	0.734	0.715
PCM—Dim 2a	−3264.72	15,597	6583.43	6695.90	6610.20	0.18 (0.15–0.20)	**0.903**	**0.922**
PCM—Dim 2b	−3448.80	15,597	6951.61	7064.07	6978.38	0.33 (0.31–0.36)	0.644	0.715
PCM—Dim 3	−3488.40	15,597	7030.81	7143.27	7057.58	0.34 (0.32–0.36)	0.639	0.711
GPCM—Dim 1	−3333.80	15,594	6727.61	6852.57	6757.35	0.26 (0.24–0.29)	0.784	0.870
GPCM—Dim 2a	−3255.57	15,593	6573.14	6702.27	6603.88	0.24 (0.21–0.26)	0.825	0.**907**
GPCM—Dim 2b	−3264.03	15,593	6590.07	6719.19	6620.80	0.20 (0.18–0.23)	0.872	0.**932**
GPCM—Dim 3	−3443.55	15,594	6947.09	7072.06	6976.84	0.34 (0.32–0.37)	0.630	0.778
GRM—Dim 1	−3325.18	15,594	6710.36	6835.33	6740.11	0.27 (0.25–0.30)	0.769	0.862
GRM—Dim 2a	−3240.87	15,593	6543.75	6672.87	6574.48	0.24 (0.21–0.26)	0.824	**0.906**
GRM—Dim 2b	−3262.08	15,593	6586.16	6715.29	6616.89	0.21 (0.19–0.24)	0.856	**0.923**
GRM—Dim 3	−3434.47	15,594	6928.94	7053.90	6958.68	0.27 (0.25–0.30)	0.768	0.861

Abbreviations: AIC, Akaike information criterion; BIC, Bayesian information criterion; CFI, comparative fit index; df, degrees of freedom; logLik, log‐likelihood; RMSEA, root mean square error of approximation; SABIC, sample‐size adjusted BIC; TLI, Tucker–Lewis index.

**TABLE 4:** Factor loadings, difficulty, and discrimination.

Item	*F*1^a^	*F*2^a^	*a*_1_	*a*_2_	*b*_1_	*b*_2_	*b*_3_	*b*_4_
Preoperative anxiety								
Item_01	0.896		3.44		−0.92	0.28	1.22	2.07
Item_02	0.922		4.06		−0.27	0.57	1.34	2.21
Item_04	0.882		3.18		−1.05	0.21	1.07	1.95
Item_05	0.905		3.63		−0.63	0.22	0.99	1.83
Need for information								
Item_03		0.875		3.08	−1.14	0.21	1.07	1.95
Item_06		0.845		2.70	−1.74	−0.83	0.26	1.55

^a^ Values below 0.2 are not shown.

Reliability was high (*r* > 0.8) for the middle area of −1.4 to +2.7 logits for anxiety and −1.4 to +1.8 logits for need for information (Figure 1D). This area covers almost the whole trait respondent's distribution for the scale need for information. For the anxiety scale the middle and upper area of respondent's trait distribution has high reliability values.

### 3.4 DIF

Significant DIF was identified for education, relationship status, number of children, whether or not the child had a surgery before, and age. The largest effect was observed for education in Item 06 (Δ*R* ^2^ = 0.037; Δ*β* = 0.028). However the magnitude of all significant DIF effects was below the predefined threshold for meaningful DIF (Tables S4–S8).

Meaningful correlations with *F*1 (perioperative anxiety) were found for state anxiety (*r* = 0.73, *p* < 0.001), state depression (*r* = 0.54, *p* < 0.001), and their subscales except for the subscale depression—euthymia (*r* = 0.32, *p* < 0.001). No meaningful correlation with *F*1 were observed for trait anxiety (*r* = 0.32, *p* < 0.001), trait depression (*r* = 0.16, *p* = 0.024), coping styles (*r* = |0.01| to 0.15, *p* = 0.038–0.950), or parental experience (*r* = 0.13, *p* = 0.009) (Table 5).

**TABLE 5:** Correlation of APAIS factors with anxiety, depression, coping styles, and parental experience regarding the health care system.

Variable	*F*1	*F*2
STADI		
Depression—euthymia—state	0.316^*^	0.114^*^
Depression—dysthymia—state	0.529^*^	0.227^*^
Depression—state	0.540^*^	0.215^*^
Agitation—state	0.638^*^	0.336^*^
Apprehension—state	0.738^*^	0.398^*^
Anxiety—state	0.733^*^	0.392^*^
STADI—state	0.726^*^	0.352^*^
Depression—euthymia—trait	0.079	0.022
Depression—dysthymia—trait	0.222^*^	0.173^*^
Depression—trait	0.164^*^	0.103
Agitation—trait	0.214^*^	0.061
Apprehension—trait	0.367^*^	0.231^*^
Anxiety—trait	0.321^*^	0.163^*^
STADI—trait	0.271^*^	0.148^*^
Coping styles (CISS)		
Task	−0.005	−0.007
Emotion	0.153^*^	0.092
Social diversion	−0.073	−0.089
Distraction	−0.038	−0.024
Avoidance	−0.068	−0.062
Parental experience		
PPE_15	0.128^*^	0.050

^*^ *p* < .05.

## 4 Discussion

The main finding of the present study is that none of the previously published factorial structure fulfilled the criteria for good model fit. Exploratory factor analysis did not identify an alternative factorial structure. The best fitting structure was the original proposed structure by Moerman et al. (1996) consisting of one scale including all four anxiety items and a second scale including the need for information items. However, it should be noted that this structure also does not exhibit a satisfactory model fit.

In contrast to the present study, many studies report excellent psychometric properties of the APAIS. These claims are primarily based on good to excellent reliability measures, assessed as internal consistency for the overall scale (Cronbach's *α* between 0.76 and 0.91 (Miraglia Raineri et al. 2019; Mohd Fahmi et al. 2015)) and for the subscales (Cronbach's *α* between 0.68 and 0.93 (Moerman et al. 1996; Nishimori et al. 2002; Mohd Fahmi et al. 2015)). In line with these findings, we found reliability values above 0.75 across a wide range of the measured latent variables, with higher values for anxiety compared to need for information. It is not surprising that higher reliability scores were found for anxiety compared to the need for information, since the former scale consists of more items. Unlike previous studies, however, the present study does not report a single reliability measure but rather takes into account that reliability varies depending on the values of the latent variables. In the range where the item difficulties fall on the scale, the scale is more informative. Consequently, the measurement error is smaller and the reliability is higher (Embretson and Reise 2000). However, this means that in areas of the scale not covered by the difficulty level of the items, precise measurements cannot be made. Therefore, these measurements are unreliable. In the present study, most participants fell within the range where reliable measurements are possible. However, when using and interpreting the APAIS, it is important to note that latent variables in individuals with very low or very high anxiety, as well as for those with very low or very high need for information, can only be assessed with limited reliability. For this reason, it is only possible to a limited extent to distinguish between different groups of people in these areas.

Nevertheless, despite the generally good reliability, other aspects—such as model fit—must also be considered when evaluating psychometric properties. The majority of published studies did not perform CFA and did not report model fit. Their conclusions are therefore primarily based on reliability measures and observed factorial structure comparable to the original proposed factorial structure. Studies that conducted CFA and reported fit indices have analyzed different factor structures. The unidimensional structure was investigated in two studies with divergent result. While Vergara‐Romero et al. (2017) found a good model fit for the unidimensional structure (RMSEA = 0.05), Maurício et al. (2021) rejected the unidimensional structure due to low model fit (RMSEA = 0.16). The original two‐dimensional structure was only supported by Wu et al. (2020) (RMSEA = 0.073) and rejected by others, which reported substantially higher RMSEA values (RMSEA from 0.16 to 0.48) (Vergara‐Romero et al. 2017; Jovanovic et al. 2022; Maurício et al. 2021). For the three‐dimensional structure (*F*1: anxiety/surgery, *F*2: anxiety/anesthesia, *F*3: need for information) also divergent results are published. Although all three studies concluded that this structure is preferable, only two showed good model fit (RMSEA = 0.069 and approximately 0.07) (Maurice‐Szamburski et al. 2013; Srinivasaiah et al. 2024), while one study did not show acceptable model fit (RMSEA = approximately 0.095) (Maurício et al. 2021). In summary, while some studies report having identified a suitable factor structure, the results are too divergent to draw definitive conclusions regarding the factor structure. This lack of consistency is not surprising, as the accuracy of parameter estimates generally improves with an increasing number of items per scale (DiStefano et al. 2019), and model fit may change accordingly, sometimes yielding better fit for shorter scales than for longer ones (Cook et al. 2009).

However, it should be noted that most studies include more than four items per scale. Moreover, as the number of items decreases, the sample size required to conduct a CFA increases. Mundfrom et al. (2005) recommend at least 320 respondents for the analysis of a two‐dimensional model with six items. Only two of the six studies that conducted CFA using APAIS data included more than 320 respondents (Vergara‐Romero et al. 2017; Jovanovic et al. 2022).

## 5 Limitations

This study has several limitations. First, participants were parents of children undergoing surgery. It cannot be ruled out that the underlying psychological construct in the assessment of anxiety related to a child's surgery is different to the assessment of anxiety related to one's own surgery. Looking on other situations associated with anxiety, comparable result for children and parent's evaluation as well as divergent results could be found. While Poole et al. (2002) reported measurement invariance in parents and children's self‐reported fear of negative evaluation and fear of positive evaluation, different factorial structure were found in parents and children for fear avoidance (Chrisman et al. 2026). Given these results, which do not necessarily indicate that the factorial structure observed in parental responses is comparable to that observed in children's responses, the significance of the present findings must, of course, be assessed accordingly. Second, larger samples are needed. Although this study included one of the largest samples of any study on the APAIS, a larger cohort would allow for more precise conclusions regarding its factor structure. Third, the highest response category was only chosen by 2.3%–9.5% of the respondents. Therefore, information for model estimation in the area of higher trait levels may be reduced. This could have influenced model fit. Samples with higher trait levels in anxiety are needed to overcome this. Fourth, this is a single‐center study in an Austrian tertiary hospital. Of course, the fact that the data were collected at only one hospital may mean that the sample is artificially homogeneous and does not reflect the full possible spectrum. It is also worth questioning how the results can be applied to other languages. A fifth limitation is that three‐quarters of the respondents were mothers. This imbalanced sex distribution may have had an influence on the results. Further aspects, which may have an influence on the results are procedure type, urgency, and severity. While procedure type was recorded the latter two were not recorded in this study. An analysis regarding procedure type was not possible due to sample size restrictions. More balanced and bigger samples are needed to better analyze these effects. Another limitation is that the APAIS consist of six items. Some studies support a unidimensional structure, others different two‐dimensional structures with two to four items in each factor. This low number of items poses a challenge for every model. From a test theory perspective, it would be desirable to have more items on each scale. However, this is not possible when reviewing an existing questionnaire with six items.

Finally, it should also be noted that the underlying research project was not primarily designed to analyze the APAIS. The APAIS was one of several questionnaires used in this project. Consequently, this study did not use a sufficient number of questionnaires to serve appropriately as an external criterion for assessing validity. Therefore, only the questionnaires used in this study could be included in the analyses.

## 6 Conclusions

In summary, the existing evidence regarding the factor structure remains inconclusive, and no definitive conclusions can yet be drawn. Nevertheless, the reliability of the APAIS in its original form appears to be satisfactory, despite the limited number of items. Further studies, including larger sample sizes and using modern psychometric methods (IRT) are needed to further explore the factorial structure of the APAIS.

## Author Contributions

**Marko Stijic**: conceptualization, data curation, Writing – review and editing, investigation. **Brigitte Messerer**: conceptualization, funding acquisition, supervision, writing – review and editing, writing – original draft. **Alexander Avian**: conceptualization, funding acquisition, formal analysis, supervision, writing – review and editing, writing – original draft, methodology, visualization, project administration. **Carmen Burger‐Ringer**: data curation, writing – review and editing, investigation.

## Funding

This study was supported by funds of the Oesterreichische Nationalbank (Anniversary Fund, project number: 18161).

## Ethics Statement

This study was performed in line with the principles of the Declaration of Helsinki. Approval was granted by the Ethics Committee of the Medical University of Graz (32‐157 ex 19/20). Informed consent was obtained from all participants involved in the study.

## Conflicts of Interest

The authors declare no conflicts of interest.

## Supporting information




**Supporting File 1**: brb371760‐sup‐0001‐SuppMat.docx

## Data Availability

The datasets used and/or analyzed during the current study are available from the corresponding author on reasonable request.
