# Supplementary material for: The Factorial Structure of the Amsterdam Preoperative Anxiety and Information Scale in Parents of Pediatric Surgical Patients: An Item Response Theory Analysis
Source: Brain Behav. 2026 Sep 14;16(9):e71760. doi: 10.1002/brb3.71760 (PMC13575621; doi:10.1002/brb3.71760)
Supplement: Supplementary file 1 [file BRB3-16-e71760-s001.docx]

**Supplemental**

Supplemental Table A.1a: unidimensional solution; (Factor loadings >0.4 are bold)

|  | *F1* |
| --- | --- |
| Item_01 | **.887** |
| Item_02 | **.915** |
| Item_03 | **.548** |
| Item_04 | **.883** |
| Item_05 | **.913** |
| Item_06 | **.530** |

Supplemental Table A.1b: two dimensional solution – confirmatory factor analysis, (Factor loadings >0.4 are bold)

|  | *F1* | *F2* |
| --- | --- | --- |
| Item_01 | **.896** | .000 |
| Item_02 | **.922** | .000 |
| Item_03 | .000 | **.875** |
| Item_04 | **.882** | .000 |
| Item_05 | **.905** | .000 |
| Item_06 | .000 | **.845** |

Supplemental Table A.1d: two dimensional solution – exploratory factor analysis; (Factor loadings >0.4 are bold)

|  | *F1* | *F2* |
| --- | --- | --- |
| Item_01 | -.069 | **.954** |
| Item_02 | -.047 | **.966** |
| Item_03 | **.650** | .180 |
| Item_04 | .054 | **.831** |
| Item_05 | .018 | **.794** |
| Item_06 | **1.00** | -.032 |

Supplemental Table A.2: Comparision of different models (likelihood ratio test or Voung test)

| *Modell* | *PCM - Dim 1* | *PCM - Dim 2a* | *PCM - Dim 2b* | *PCM - Dim 3* | *GPCM - Dim 1* | *GPCM - Dim 2a* | *GPCM - Dim 2b* | *GPCM - Dim 3* | *GRM - Dim 1* | *GRM - Dim 2a* | *GRM - Dim 2b* | *GRM - Dim 3* |
| --- | --- | --- | --- | --- | --- | --- | --- | --- | --- | --- | --- | --- |
| PCM - Dim 1 |  | <.001 | .038 | NaN | <.001 |  |  |  | .069 |  |  |  |
| PCM - Dim 2a | <.001 |  | .622 | <.001 |  | .001 |  |  |  | .936 |  |  |
| PCM - Dim 2b | .038 | .622 |  | .266 |  |  | <.001 |  |  |  | .562 |  |
| PCM - Dim 3 | NaN | <.001 | .266 |  |  |  |  | <.001 |  |  |  | .867 |
| GPCM - Dim 1 | <.001 |  |  |  |  | <.001 | <.001 | NaN | .043 |  |  |  |
| GPCM - Dim 2a |  | .001 |  |  | <.001 |  | .245 | <.001 |  | .002 |  |  |
| GPCM - Dim 2b |  |  | <.001 |  | <.001 | .245 |  | .08 |  |  | .324 |  |
| GPCM - Dim 3 |  |  |  | <.001 | NaN | <.001 | .08 |  |  |  |  | .005 |
| GRM - Dim 1 | .069 |  |  |  | .043 |  |  |  |  | <.001 | <.001 | .002 |
| GRM - Dim 2a |  | .936 |  |  |  | .002 |  |  | <.001 |  | .293 | <.001 |
| GRM - Dim 2b |  |  | .562 |  |  |  | .324 |  | <.001 | .293 |  | <.001 |
| GRM - Dim 3 |  |  |  | .867 |  |  |  | .005 | .002 | <.001 | <.001 |  |

Supplemental Table A.3 Measurement invariance according to sex

|  | overall test of total DIF effect (1-3) | | | | uniform DIF (1-2) | | | | | non uniform DIF (2-3) | | | |
| --- | --- | --- | --- | --- | --- | --- | --- | --- | --- | --- | --- | --- | --- |
|  | ΔR² | | | p-value | ΔR² | | | Δβ | p-value | ΔR² | | | p-value |
|  | McFadden | Nagelkerke | Cox Snell |  | McFadden | Nagelkerke | Cox Snell |  |  | McFadden | Nagelkerke | Cox Snell |  |
| I_01 | <.001 | <.001 | <.001 | .851 | <.001 | <.001 | <.001 | .004 | .664 | <.001 | <.001 | <.001 | .714 |
| I_02 | .001 | .001 | .001 | .628 | <.001 | <.001 | <.001 | .001 | .852 | .00 | .001 | .001 | .344 |
| I_03 | .001 | .002 | .001 | .650 | <.001 | .001 | .001 | .012 | .457 | <.001 | .001 | .001 | .578 |
| I_04 | .001 | .001 | .001 | .574 | .001 | <.001 | <.001 | .003 | .388 | <.001 | <.001 | <.001 | .545 |
| I_05 | .001 | <.001 | <.001 | .669 | <.001 | <.001 | <.001 | .001 | .866 | .001 | <.001 | <.001 | .379 |
| I_06 | <.001 | <.001 | <.001 | .965 | <.001 | <.001 | <.001 | -004 | .811 | <.001 | <.001 | <.001 | .905 |

Supplemental Table A.4: Measurement invariance according to education (high school degree y/n)

|  | overall test of total DIF effect (1-3) | | | | uniform DIF (1-2) | | | | | non uniform DIF (2-3) | | | |
| --- | --- | --- | --- | --- | --- | --- | --- | --- | --- | --- | --- | --- | --- |
|  | ΔR² | | | p-value | ΔR² | | | Δβ | p-value | ΔR² | | | p-value |
|  | McFadden | Nagelkerke | Cox Snell |  | McFadden | Nagelkerke | Cox Snell |  |  | McFadden | Nagelkerke | Cox Snell |  |
| I_01 | <.001 | <.001 | <.001 | .919 | <.001 | <.001 | <.001 | <.001 | .881 | <.001 | <.001 | <.001 | .703 |
| I_02 | -003 | .002 | .001 | .190 | <.001 | <.001 | <.001 | .002 | .507 | .002 | .001 | .001 | .089 |
| I_03 | .008 | .018 | .017 | .005 | .004 | .010 | .009 | .013 | .016 | .004 | .008 | .008 | .026 |
| I_04 | .025 | .018 | .017 | <.001 | .006 | .005 | .005 | .009 | .006 | .018 | .014 | .013 | <.001 |
| I_05 | .014 | .009 | .009 | <.001 | .007 | .005 | .004 | .013 | .002 | .007 | .005 | .004 | .002 |
| I_06 | .016 | .037 | .035 | <.001 | .012 | .026 | .025 | .028 | <.001 | .005 | .011 | .010 | .012 |

Supplemental Table A.5: Measurement invariance according to relationship status (relationship yes: married, partner/no: no partner, divorced, widowed )

|  | overall test of total DIF effect (1-3) | | | | uniform DIF (1-2) | | | | | non uniform DIF (2-3) | | | |
| --- | --- | --- | --- | --- | --- | --- | --- | --- | --- | --- | --- | --- | --- |
|  | ΔR² | | | p-value | ΔR² | | | Δβ | p-value | ΔR² | | | p-value |
|  | McFadden | Nagelkerke | Cox Snell |  | McFadden | Nagelkerke | Cox Snell |  |  | McFadden | Nagelkerke | Cox Snell |  |
| I_01 | .017 | .014 | .013 | <.001 | .016 | .013 | .012 | .026 | <.001 | .002 | .001 | .001 | .215 |
| I_02 | .006 | .004 | .004 | .021 | .001 | .001 | .001 | .001 | .306 | .006 | .004 | .003 | .010 |
| I_03 | .001 | .001 | .001 | .700 | <.001 | .001 | .001 | .003 | .509 | <.001 | <.001 | <.001 | .598 |
| I_04 | .003 | .002 | .002 | .099 | .001 | .001 | .001 | .001 | .190 | .002 | .002 | .001 | .089 |
| I_05 | <.001 | <.001 | <.001 | .966 | <.001 | <.001 | <.001 | .001 | .811 | <.001 | <.001 | <.001 | .912 |
| I_06 | .004 | .009 | .009 | .059 | <.001 | <.001 | <.001 | .001 | .839 | .004 | .009 | .009 | .018 |

Supplemental Table A.6: Measurement invariance according to number of children (1 vs. ≥2)

|  | overall test of total DIF effect (1-3) | | | | uniform DIF (1-2) | | | | | non uniform DIF (2-3) | | | |
| --- | --- | --- | --- | --- | --- | --- | --- | --- | --- | --- | --- | --- | --- |
|  | ΔR² | | | p-value | ΔR² | | | Δβ | p-value | ΔR² | | | p-value |
|  | McFadden | Nagelkerke | Cox Snell |  | McFadden | Nagelkerke | Cox Snell |  |  | McFadden | Nagelkerke | Cox Snell |  |
| I_01 | <.001 | <.001 | <.001 | .902 | <.001 | <.001 | <.001 | <.001 | .994 | <.001 | <.001 | <.001 | .649 |
| I_02 | <.001 | <.001 | <.001 | .869 | <.001 | <.001 | <.001 | .001 | .863 | <.001 | <.001 | <.001 | .617 |
| I_03 | .001 | .001 | .001 | .710 | <.001 | <.001 | <.001 | .002 | .720 | <.001 | .001 | .001 | .456 |
| I_04 | .003 | .003 | .003 | .098 | .002 | .001 | .001 | .003 | .136 | .002 | .001 | .001 | .120 |
| I_05 | .004 | .003 | .003 | .068 | <.001 | <.001 | <.001 | .001 | .539 | .004 | .003 | .002 | .025 |
| I_06 | .001 | .003 | .003 | .488 | .001 | .002 | .002 | .005 | .317 | <.001 | .001 | .001 | .511 |

Supplemental Table A.7: Measurement invariance according to the fact child had a surgery before (yes / no)

|  | overall test of total DIF effect (1-3) | | | | uniform DIF (1-2) | | | | | non uniform DIF (2-3) | | | |
| --- | --- | --- | --- | --- | --- | --- | --- | --- | --- | --- | --- | --- | --- |
|  | ΔR² | | | p-value | ΔR² | | | Δβ | p-value | ΔR² | | | p-value |
|  | McFadden | Nagelkerke | Cox Snell |  | McFadden | Nagelkerke | Cox Snell |  |  | McFadden | Nagelkerke | Cox Snell |  |
| I_01 | .011 | .008 | .008 | .001 | .010 | .008 | .007 | .020 | <.001 | .001 | <.001 | <.001 | .441 |
| I_02 | .001 | <.001 | <.001 | .737 | .001 | <.001 | <.001 | <.001 | .438 | <.001 | <.001 | <.001 | .925 |
| I_03 | <.001 | <.001 | <.001 | .893 | <.001 | <.001 | <.001 | .002 | .633 | <.001 | <.001 | <.001 | .994 |
| I_04 | .008 | .006 | .006 | .004 | .001 | .001 | .001 | <.001 | .256 | .007 | .005 | .005 | .002 |
| I_05 | <.001 | <.001 | <.001 | .844 | <.001 | <.001 | <.001 | <.001 | .582 | <.001 | <.001 | <.001 | .848 |
| I_06 | <.001 | .001 | .001 | .808 | <.001 | <.001 | <.001 | .001 | .791 | <.001 | .006 | .001 | .551 |

Supplemental Table A.8: Measurement invariance according to age (≤ 40 years / > 40 years)

|  | overall test of total DIF effect (1-3) | | | | uniform DIF (1-2) | | | | | non uniform DIF (2-3) | | | |
| --- | --- | --- | --- | --- | --- | --- | --- | --- | --- | --- | --- | --- | --- |
|  | ΔR² | | | p-value | ΔR² | | | Δβ | p-value | ΔR² | | | p-value |
|  | McFadden | Nagelkerke | Cox Snell |  | McFadden | Nagelkerke | Cox Snell |  |  | McFadden | Nagelkerke | Cox Snell |  |
| I_01 | .004 | .003 | .003 | .118 | .003 | .002 | .002 | .002 | .083 | .001 | .001 | .001 | .260 |
| I_02 | <.001 | <.001 | <.001 | .849 | <.001 | <.001 | <.001 | .003 | .605 | <.001 | <.001 | <.001 | .807 |
| I_03 | .005 | .011 | .011 | .037 | .002 | .004 | .004 | .018 | .141 | .003 | .008 | .007 | .036 |
| I_04 | .002 | .002 | .002 | .229 | .001 | .001 | .001 | .002 | .236 | .001 | .001 | .001 | .215 |
| I_05 | .001 | <.001 | <.001 | .695 | <.001 | <.001 | <.001 | .001 | .668 | <.001 | <.001 | <.001 | .461 |
| I_06 | .005 | .011 | .010 | .047 | .001 | .001 | .001 | .011 | .422 | .004 | .010 | .009 | .019 |
